# The Relationship between Power Generated by Thrust and Power to Overcome Drag in Elite Short Distance Swimmers

**DOI:** 10.1371/journal.pone.0162387

**Published:** 2016-09-21

**Authors:** Giorgio Gatta, Matteo Cortesi, Paola Zamparo

**Affiliations:** 1 Department for Life Quality Studies, Rimini, School of Pharmacy, Biotechnology and Sport Science, University of Bologna, Bologna, Italy; 2 Department of Neurological, Biomedical and Movement Sciences, University of Verona, Verona, Italy; Universitat Zurich, SWITZERLAND

## Abstract

At constant average speed (v), a balance between thrust force (Ft) and drag force (Fd) should occur: Ft−Fd = 0; hence the power generated by thrust forces (Pt = Ft·v) should be equal to the power needed to overcome drag forces at that speed (Pd = Fd·v); the aim of this study was to measure Pt (tethered swims), to estimate Pd in active conditions (at sprint speed) and to compare these values. 10 front crawl male elite swimmers (expertise: 93.1 ± 2.4% of 50 m world record) participated to the study; their sprint speed was measured during a 30 m maximal trial. Ft was assessed during a 15 s tethered effort; passive towing measurement were performed to determine speed specific drag in passive conditions (k_P_ = passive drag force/v^2^); drag force in active conditions (Fd = k_A_·v^2^) was calculated assuming that k_A_ = 1.5·k_P_. Average sprint speed was 2.20 ± 0.07 m·s^-1^; k_A_, at this speed, was 37.2 ± 2.7 N·s^2^·m^-2^. No significant differences (paired t-test: p > 0.8) were observed between Pt (399 ± 56 W) and Pd (400 ± 57 W) and a strong correlation (R = 0.95, p < 0.001) was observed between these two parameters. The Bland-Altman plot indicated a good agreement and a small, acceptable, error (bias: -0.89 W, limits of agreement: -25.5 and 23.7 W). Power thrust experiments can thus be suggested as a valid tool for estimating a swimmer’s power propulsion.

## Introduction

When a swimmer moves in water he/she generates propulsion through the action of the upper and lower limbs; the application of these forces is not constant during a cycle (as in all cyclic forms of locomotion [1]), but with larger propulsive forces higher speeds can be reached. When a swimmer moves in water he/she is also subjected to resistive forces (hydrodynamic resistance, drag) that are larger the higher the swimming speed. The swimmer's velocity thus shows intra-cyclic variations that are the result of intra-cyclic phases of acceleration (limbs thrust) and deceleration (water drag) [2]. These fluctuations in speed depend on the swimming stroke: in front crawl and backstroke are much lower than in breaststroke and butterfly (15–20% vs. 45–50%) [3].

During a race, a swimmer must be able to minimize the time spent in the starting phase, in the turning phases and in the stroking phases. In the start and turn phases the speed is larger than in the stroking phases due to the possibility to exert additional propulsive forces against the blocks/pool wall and to minimize water resistance (e.g. by gliding underwater) [4]. In these phases of the race the biomechanical determinants of performance are somewhat different than in the stroking phases; they will not be considered in this study.

The speed attained in the stroking phases (neglecting start and turns) is termed clean swimming speed and, during a race (or a lap), is almost constant [5]; indeed, was the swimmer able to produce (average) propulsive forces larger than the (average) drag forces he/she experiences he/she would accelerate; indeed, maximal (clean) swimming speed is attained when a swimmer uses his/her maximal propulsive force for progression.

Thus, at maximal (clean) swimming speed (as well as in all conditions where average speed is constant and acceleration is nil) a balance between thrust force (Ft) and drag force (Fd) should occur: Ft−Fd = 0, as suggested by Toussaint et al. [5]. Because power is the product of force and velocity [6], at constant (maximal) speed the power required to overcome drag (Pd = Fd·v) and the power required to push the swimmer forward (Pt = Ft·v) should be equal [5].

This state of affairs implies that, to improve performance in the stroking phase of the race, a swimmer can either increase his/her propulsive forces (e.g. with a proper training) or reduce his/her hydrodynamic resistance (e. g. by improving his/her swimming technique or his/her hydrodynamic body position in water), or both. As an example, rubber full body swimsuits allow a swimmer to reach larger speeds since, for a given power thrust (a which characterizes a given swimmer), the power needed to overcome drag forces can be reduced [7].

These principles and considerations hold in all cyclic forms of locomotion; as an example, in cycling power output can be calculated either based on the resistive forces that the cyclist has to overcome (e. g. Pd: rolling and air resistance, on flat terrain [8]) or based on the propulsive forces he/she generates on the pedals (e. g. Pt [9]).

In water, however, the “equal power assumption” (Pd = Pt) is difficult to demonstrate; this because it is quite difficult to measure forces in the aquatic environment.

To estimate the trust force (Ft), Toussaint et al. [5] proposed the use of a system (MAD system) that consists of an integrated series of anchored paddles. The swimmer pulls the paddle and pushes his body forward; the propulsion force is measured by means of force transducers mounted on the paddles. The major criticism of this method in measuring accurately Ft concerns the use of arms alone without considering the propulsion of the legs; thus the values of Ft are necessarily underestimated. Other researchers investigated the hand position during the pull phase of the stroke to determine the net force produced by the hand and the relative contribution of lift and drag vectors [10]. However, a direct assessment of the swimmer’s propulsion during hand movements remains complicated, and the propulsive force in swimming is still difficult to quantify [11].

With the objective of solving this issue, the measurement of the pulling force during tethered or semi-tethered swimming was proposed as an alternative tool for evaluating Ft [12–13–14–15–16]. With this method a load cell is anchored to the pool wall and connected to the swimmer’s belt through a rope; in the case of the tethered experiments there is no forward displacement of the swimmer (pulling force is assessed at zero forward speed). Despite the different reports in the literature about this method, after considering its advantages (reliable and user-friendly) and disadvantages (for some authors the transferability to real swimming is debatable), tethered swimming is the most frequently used method for determining the biomechanics profile of a swimmer [14–17]. A strong relationship between tethered force and swimmer's speed was indeed reported by several authors [14–18] even if the only reliable parameter for evaluating this relationship seems to be the average force of the all-out tethered [19]. The average force measured by the tethered method should then correspond to the useful, average, force to overcome water resistance at maximum speed [18–20].

Estimating the drag force (Fd) by means of passive towing experiments necessarily leads to an underestimation of the power needed to overcome drag forces since passive drag is lower than active drag [21]. Thus, several studies have attempted to measure the swimmer’s Fd in active conditions [22–23–24–25–26]. Furthermore, in recent years, there have been several attempts to estimate Fd by applying the computational fluid dynamics simulation to a swimmer’s flow [27]. The scientific discussion about the best method to assess active drag remains a controversial issue within the scientific community [28]. Although the swimmer’s movements during active propulsion create additional drag [29], the hydrodynamic resistance created when towing a swimmer in a hydrodynamic stable position has shown to be a remarkably consistent method for measuring the swimmer’s passive drag [30]. To estimate the active drag from passive towing, a procedure was proposed by Gatta et al. [21]: in that study it was shown, by means of a planimetric method, that frontal area during a swimming stroke is 1.5 larger than that observed during passive towing (in the front crawl) so that the former can be calculated based on the latter.

To the best of our knowledge, the assumption whether the thrust force should be equal to the useful force to overcome the swimmer’s drag at maximal swimming speed has never been tested in the scientific literature mainly because both parameters are quite difficult to assess. The aim of this study was to verify whether there is a balance between the power generated by thrust forces and the power needed to overcome drag forces in front crawl swimming by using a tethered test to assess Ft (and Pt) and by estimating active drag (Fd, and hence Pd) based on measures of passive drag (at maximal speed). We performed the experiments with the front crawl since its one of the strokes with the smallest intra-cyclic variations in speed and we decided to perform the experiments at maximal swimming speed to simulate the clean swimming speed during the stroking phase of a sprint race.

## Materials and Methods

### Participants

Ten freestyle high-level male swimmers participated in this study (23.5 ± 3.4 years of age, 1.88 ± 0.06 m of stature, 80.8 ± 8.98 kg of body mass and 55 ± 10 km/week of training volume); long-course 50-m and 100-m freestyle personal best times are 22.5 ± 0.6 s and 49.5 ± 1.4 s, respectively (representing 93 ± 2 and 95 ± 3% of the World Record). The experiments were performed during the autumn of 2015, when the swimmers were in their competition period. All participants were non-smokers, and none of them was following specific dietary interventions. All procedures described here were approved by the Bioethics Committee of the University of Bologna. Written informed consent was obtained from all participants prior to their voluntarily participation in the study.

### Design and Methodology

The test sessions were conducted in a 50 m indoor swimming pool (average water temperature: 28.0 ± 0.5°C), and the testing procedure was completed within 3 hours for each swimmer. To measure the swimmer’s maximal speed (V_30_), each participant completed 3 maximal effort front crawl trials of 35 m from a push start. Before these trials, the participants performed a 20 min warm-up period. Average (maximal) speed was calculated between the 10^th^ and 40^th^ m from the start by determining the time it took for the participant’s head to pass between two points that were 30 m apart. The time was measured by means of two aligned underwater cameras (TS-6021PSC, Sony Hyper Had, Tokyo, Japan, sampling rate: 25 Hz) placed at the end points of the testing zone; the cameras were synchronized using a specially developed software application [31]. Participants were instructed to not take a breath through the 30 m test section of the 50 m pool. The best trial (largest value of V_30_ for each swimmer) was considered in further analysis.

To assess Ft, 3 trials of 15 s at maximal intensity during tethered front crawl swimming (without breathing) were executed. For the present study, the authors selected the tethered test protocol of 15 s that replicates an equal duration of the maximal speed trial in free swimming used in this study [17]. The testing apparatus consisted of a load-cell system (range 0–2500 N, sampling rate 1000 Hz, Globus Ergometer, Globus^™^, Codognè, Italy) that was connected through a non-elastic cable to a Globus Ergometer data acquisition system. The load-cell was anchored to a fixed starting block by a steel cable perpendicularly to swimmer direction, and the participants were connected to the load cell by means of a nylon belt. The swimmers adopted a horizontal position with the cable full extended before the starting signal. Data collection only started after the first stroke cycle was performed and the trial’s end was highlighted by an acoustic signal. The load-cell accuracy was tested by means of a static calibration with masses of 1–5–10–20 kg. The signal from the load cell was amplified using a Globus amplifier (Tesys 400, Globus^™^, Codogne, Italy) and fed through an analog-to-digital converter (12 bit). Dynamometrical data were recorded and processed with Globus Ergometer software and were then exported to a PC as TXT format data. The best trial (the largest Ft value recorded for each swimmer) was considered in further analysis.

The swimmers’ passive drag (Fd) was measured using an electro-mechanical device (Swim-Spektro, Talamonti Spa, Ascoli Piceno, Italy). A low voltage isokinetic engine was positioned at the edge of the pool and measured the force (N) needed for the swimmer’s tow [32]. The device was calibrated before each session. After the experiments, data were downloaded to a PC and further analysed using dedicated software (DB:4, Talamonti Spa). Each swimmer was connected to the machine via a non-elastic wire and was dragged at a constant velocity (1.0, 1.3, 1.6, 1.9 and 2.2 m·s^-1^). The participants performed the passive towing test using the best glide position following the same protocol used in a previous study [32] as follows: the swimmer’s hands were held together with the swimmer’s head between the arms extended overhead, while the lower limbs and feet were in maximum extension.

The average values of towing force (Fd, N) were computed between the 10^th^ and 20^th^ m from the starting wall, when stabile data were attained. Three trials were performed for each swimmer and each velocity but only data referring to the largest speed (2.2 m·s^-1^) were considered in further analysis. Also in this case, the best trial (with the lowest values of Fd, i.e. with the best glide position for each swimmer) was selected. These values of Fd were divided by the square of the corresponding speed (2.2 m·s^-1^) to obtain the speed-specific drag (k_P_ = Fd /v^2^, N·s^2^·m^-2^). As proposed by Gatta et al. [21] speed specific drag in active conditions (k_A_) was then calculated by multiplying k_P_ by 1.5 which is a factor that takes into account that average frontal area during a swimming cycle is 1.5 times larger than during passive towing (in the front crawl).

Before both experiments, and with the aim of familiarizing the swimmers with the methodology, several training sessions were conducted at various intensities and with different durations.

For all participants the three tests were performed in the following order: 1—maximal speed test; 2 –test to determine the power needed to overcome drag forces; and 3 –test to determine the power generated by thrust forces. The first and third tests were separated by the second test to prevent any effect of fatigue, and each trial was separated by a minimum of 20 min of active recovery.

The following parameters were thus calculated for each swimmer:

- Mean swimming speed (V_30_) during the maximal swimming test and stroke frequency values (SFv) during this test;
- Mean thrust force (Ft) as the average force registered during the 15 s of tethered swimming and stroke frequency values (SFt) during this test;
- Mean thrust power (Pt) as a product of Ft and V_30_;
- Speed specific drag in passive (k_P_) and active conditions (k_A_) as described above [17]
- Mean drag power in passive (Pd _P_ = k_P_ V_30_^3^) and active (Pd _A =_ k_A_ V_30_^3^) conditions.

### Statistical analysis

Data presented as mean ± SD. To quantify the agreement between the 3 trials (for the Ft and Fd experiments), the coefficient of variation (CV%) was calculated. Correlation between variables in linear regression was evaluated as indicated by Geigy Scientific Tables. A paired t-test was used to investigate eventual differences in the variables measured with different approaches (velocity, stroke frequency and power). A Shapiro-Wilk test was performed for the evaluation of normality for statistical distribution. A Bland-Altmann plot [33] was constructed to evaluate the agreement between Pt and Pd values; the upper and lower limits of agreement were also calculated for these data. Statistical analysis was performed using SPSS for Windows (SPSS Statistic 17.0).

## Results

Only the “best” trials (the highest Ft and the lowest Fd values for each swimmer) were utilized in this study to calculate Pd and Pt. Considering all trials the coefficient of variation (CV%) amounted to 3.5 ± 1.9% for Fd and 6.2 ± 1.7% for Ft.

The individual values of maximal swimming speed (V_30_), stroke frequency (SFv) and length (SLv) during the maximal swim trials are reported in Table 1 along with the average and maximal values of tethered force (Ft), of stroke frequency during this test (SFt) and of power generated by thrust force (Pt). The values of drag force (Fd) as determined during the towing tests at different speeds (1–2.2 m·s^-1^) are reported in Table 2 along with the coefficient of drag in passive (k_P_) and active (k_A_) conditions (at a speed of 2.2 m·s^-1^) and of power needed to overcome drag forces (Pd) in passive and active conditions. No significant differences were observed between SFt and SFv (paired t-test: p = 0.247) as well as between Pt and Pd (paired t-test: p = 0.828); moreover, maximal swimming speed (2.2 ± 0.07 m·s^-1^) was similar to the maximal speed during passive drag measurements (2.2 m·s^-1^).

**Table 1:** Individual data assessed during the maximal swim trial and during the tethered test.

	V_30_ (m·s^-1^)	SFv (Hz)	SLv (m)	Ft mean (N)	Ft max (N)	SFt (Hz)	Pt (W)
**1**	2.13	0.92	2.32	170	303	0.99	362
**2**	2.30	1.10	2.10	210	507	1.08	483
**3**	2.21	1.01	2.21	182	376	0.88	401
**4**	2.17	0.92	2.36	179	429	0.94	389
**5**	2.25	0.92	2.27	201	396	0.83	453
**6**	2.13	1.05	2.04	141	235	1.03	301
**7**	2.21	0.97	2.29	181	296	0.83	401
**8**	2.30	0.98	2.36	191	336	0.98	438
**9**	2.13	0.94	2.29	156	370	0.87	333
**10**	2.21	0.93	2.39	195	319	1.01	432
**mean**	2.20	0.97	2.26	181	357	0.94	399
**SD**	0.07	0.06	0.11	21	77	0.09	56

V_30_: average speed during the maximal swim test; SFv: stroke frequency during the maximal swim test; SLv: stroke length during the maximal swim test; Ft: tethered force (mean and max values); SFt: stroke frequency during the tethered test; Pt = Ft· V_30_.

**Table 2:** Individual data assessed during passive drag measurements.

	Fd (N) 1.0 m·s^-1^	Fd (N) 1.3 m·s^-1^	Fd (N) 1.6 m·s^-1^	Fd (N) 1.9 m·s^-1^	Fd (N) 2.2 m·s^-1^	k_P_ 2.2 m·s^-1^	k_A_ 2.2 m·s^-1^	Pd_P_ (W)	Pd_A_ (W)
**1**	31.5	40.2	56.0	77.7	115.6	23.9	35.9	231	346
**2**	37.2	46.3	66.0	88.0	130.0	26.9	40.4	327	491
**3**	30.8	44.4	58.8	75.8	116.5	24.1	36.2	260	390
**4**	34.2	47.0	64.0	82.0	128.1	26.5	39.8	270	406
**5**	32.3	42.3	58.2	92.2	126.0	26.0	39.0	297	444
**6**	29.6	44.0	49.8	71.8	100.3	20.7	31.1	200	300
**7**	29.0	45.4	62.1	86.0	121.7	25.1	37.7	271	406
**8**	32.7	48.3	58.8	87.8	120.3	24.9	37.4	302	454
**9**	30.0	43.0	57.4	76.0	115.4	23.8	35.7	230	345
**10**	34.1	46.1	61.8	86.9	125.1	25.8	38.7	279	418
**mean**	32.1	44.7	59.3	82.4	119.9	24.8	37.2	267	400
**SD**	2.5	2.4	4.6	6.7	8.6	1.8	2.7	38	57

Fd: average force during passive drag measurements; k_P_: speed specific drag in passive conditions (at a speed of 2.2 m·s^-1^); k_A_: speed specific drag in active conditions (at a speed of 2.2 m·s^-1^: k_A_ = 1.5· k_P_); Pd_P_ = k_P_· V_30_^3^; Pd_A_ = k_A_· V_30_^3^.

The relationship between maximal swimming speed and tethered force is significant when the average values of Ft are considered (V_30_ = 1.72 + 0.0027· Ft mean; R = 0.820, p < 0.01) but not when the maximal values of Ft are considered (V_30_ = 2.04 + 0.0004· Ft max; R^2^ = 0.519, NS).

**Fig 1:**
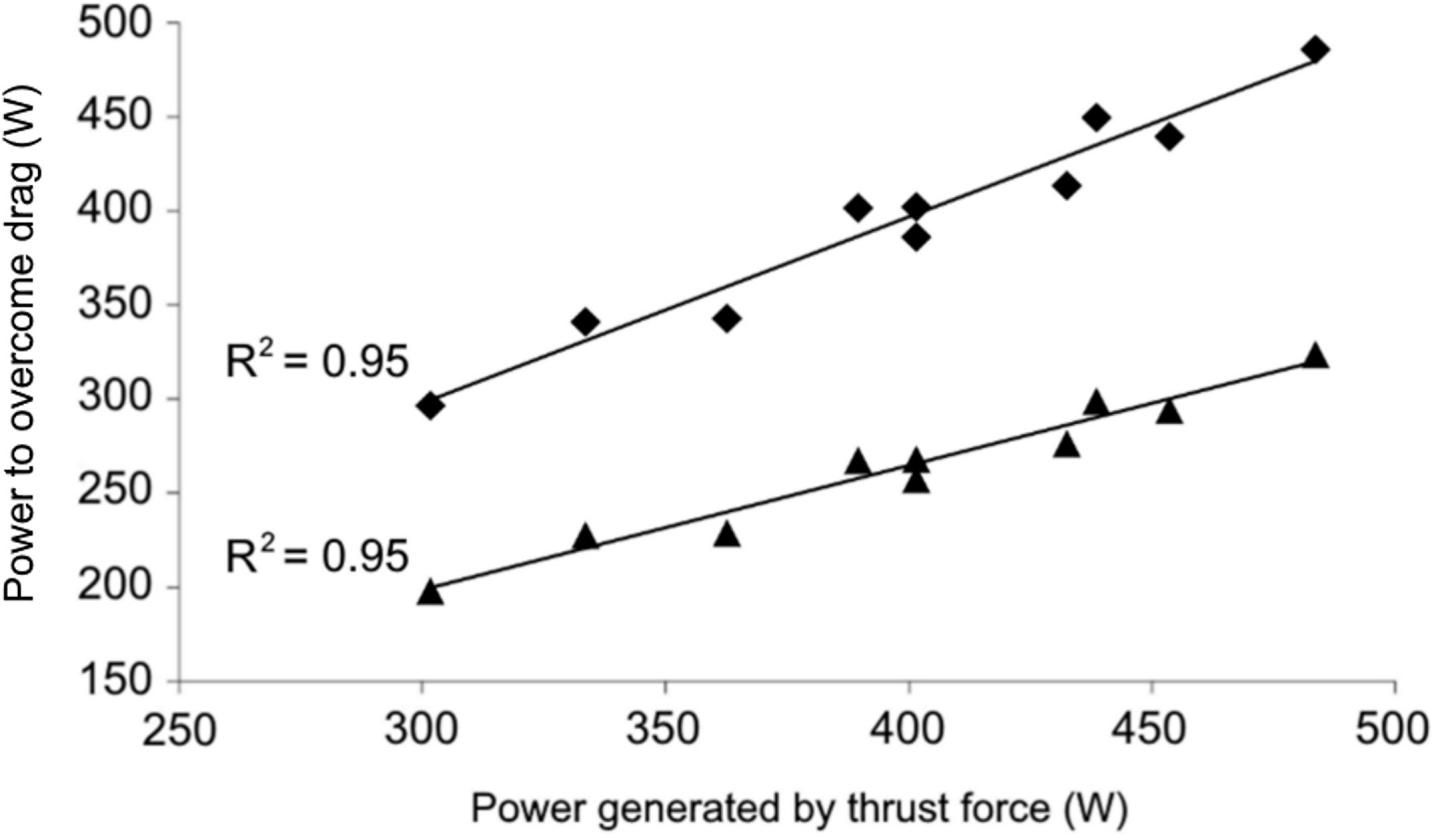
The relationships between power generated by thrust forces (Pt) and power needed to overcome drag forces (Pd_A_: active and Pd_P_: passive). Pd_P_ = 1.68 + 0.664·Pt, R^2^ = 0.953, N = 10, p < 0.001 (triangles); Pd_A_ = 1.09 + 0.999·Pt, R^2^ = 0.952, N = 10, p < 0.001 (diamonds).

The relationship between Pt and Pd is reported in Fig 1; this relationship is significant (p < 0.001) considering both the passive (triangles: Pd_P_) and active (diamonds: Pd_A_) values of power needed to overcome drag forces; however, the slope of this relationship is much closer to the identity line for Pd_A_ (0.999) than for Pd_P_ (0.664).

**Fig 2:**
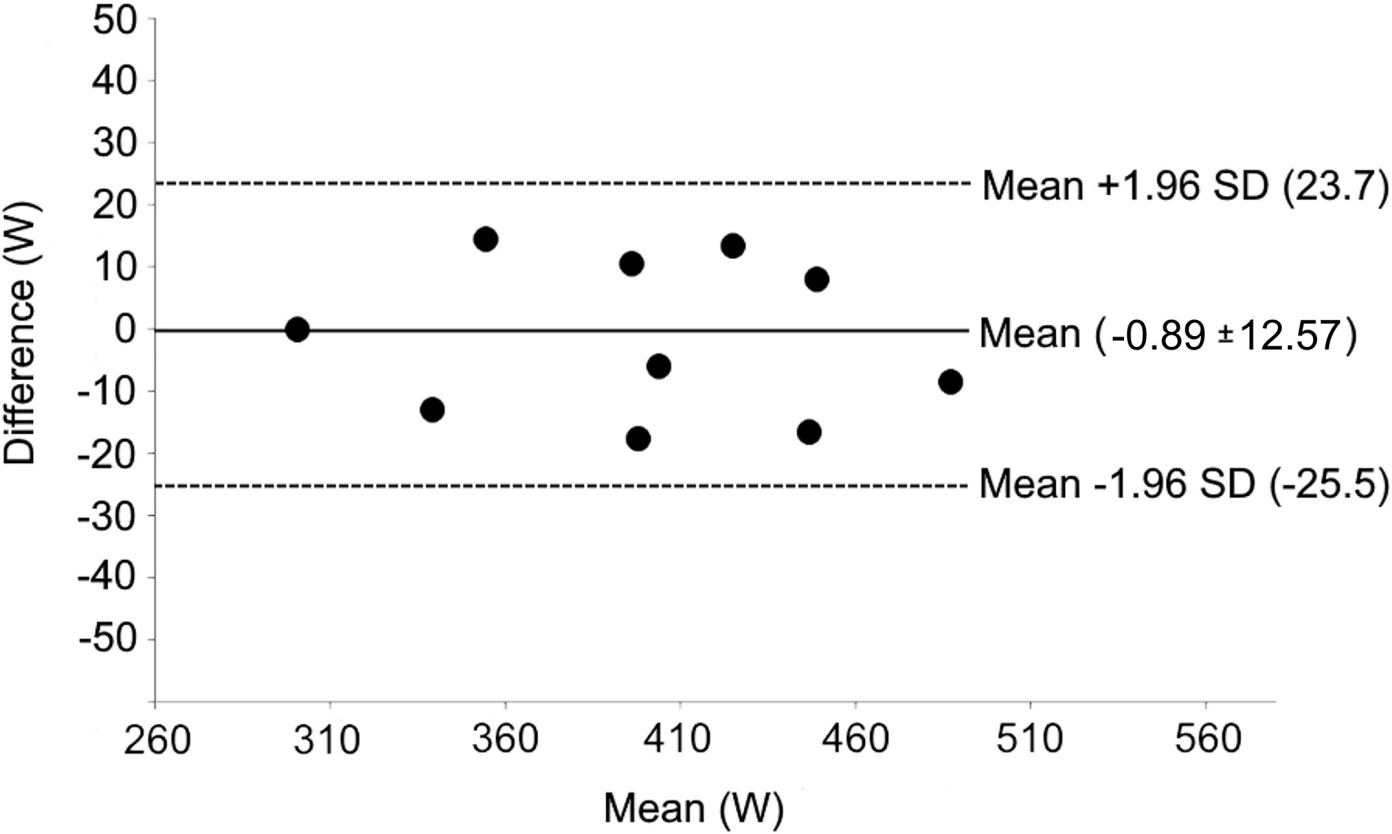
Bland-Altman plot of the differences between Pt and Pd (in active conditions) as a function of the corresponding mean. The dotted and solid lines represent, respectively, the ± 1.96 SD limits of agreement and the mean.

In Fig 2 the Bland Altman plot is reported; this figure indicates that the values of power generated by thrust forces (Pt) are in good agreement with those of power needed to overcome drag force (Pd_A_). The bias amounts to -0.89 W and the levels of agreement are -25.5 and 23.7 W.

## Discussion

The aim of this study was to estimate the power generated by thrust forces and the power needed to overcome drag force in front crawl sprint swimmers in order to investigate the balance, at a given speed (2.2 ± 0.07 m· s^-1^, in this study), between thrust force (Ft) and drag force (Fd). We used the tethered test to compute the thrust force and we calculated the active drag force from passive drag experiments. Data reported in this study show that a good (significant) relationship indeed exists between the power generated by thrust forces (Pt = 399 ± 56 W) and the power needed to overcome drag force and that this relationship is closer to the identity line when power drag in active conditions (Pd_A_: 400 ± 57 W) is considered rather than in passive conditions (Pd_P_ = 267 ± 38 W).

The individual differences between Pd_A_ and Pt (on average = -0.89 ± 12.57 W) can not be attributed to individual differences between V_30_ and the velocity at which K_P_ was assessed (on average = 0.004 ± 0.065 m·s^-1^) neither between SFt and SFv (on average = 0.03 ± 0.08 Hz) since no relationship is observed when the individual differences in velocity or SF are plotted against the differences in power output (R^2^ = 0.018 and 0.045, respectively); they can be attributed to an incorrect determination of Ft and/or Fd (measurement errors and intra-individual variability) as discussed in detail below.

1. **Test duration and swimming speed.** The speed of the maximal swim test was calculated over the last 30 m of a 35 m maximal sprint; the time to cover this distance was thus comparable to that of the tethered test (15 s): 2.2 m·s^-1^ in 30 m = 13.6 s. The swimmers, however, had to accelerate to that speed from zero (a push start) in the first 5 m and, also in this phase, they generated thrust; the duration of the entire swim test is, however, as close as possible, to that of the tethered test.
2. **Tethered Force.** Many authors have investigated the tethered force using different trial durations, ranging from a few seconds [13–34] to several minutes [12]. The average tethered force measured in this study was 181 ± 21 N for a 15 s front crawl trial, e.g. comparable to the values reported by Mosterd et al. [35] and Morouco et al. [17]: 132, 113 and 101 N in tethered trials of 20, 30 and 60 s, respectively. The difference between our data and their values could be attributed to the age and skill level of the swimmers.
3. **Drag force.** The average speed-specific passive drag measured in this study (k_P_) on male athletes was 24.8 N·s^2^·m^-2^, which is similar to the values reported by Cortesi et al. [7] and Zamparo et al. [26]. The power needed to overcome drag forces was calculated by considering that k_A_ = 1.5 k_P_ in the front crawl (Gatta et al. [21]); k_A_ was then calculated based on individual values of k_P_ but assuming a factor = 1.5 for all subjects. This factor represents the average difference between active and passive frontal area as measured in the study of Gatta et al. [21] in male and female competitive swimmers and thus does not allow to take into account inter-individual differences in “active” frontal area that could indeed contribute to the inter-subject differences between Pt and Pd_A_. The choice not to use a direct method for measuring active drag can be considered a limitation of this study. However, the active drag measure remains controversial within the scientific community and we used a procedure that allows indeed to estimate the power needed to overcome drag force with quite a good accuracy (see Figs 1 and 2).
4. **Best vs. average values**. Whereas the largest values of Ft (for each swimmer) were utilized in this study to calculate Pt (i.e. the best value in the three trials), the lowest values of k_P_ were selected to estimate k_A_, and then Pd_A_. Indeed, the “best trial” in passive towing experiments is the one where the lowest value of Fd is recorded, larger values being attributable to a non optimal hydrodynamic position of the swimmer during the test or to a incorrect test execution. When the average (instead of the best) values are considered, the relationships between the power generated by thrust force (Pt) and the power needed to overcome drag force (Pd_A_: active and Pd_P_: passive) are still highly significant: Pd_P_ = 38.69 + 0.618 Pt, R^2^ = 0.942, N = 10, p < 0.001; Pd_A_ = 58.04 + 0.926 Pt, R^2^ = 0.942, N = 10, p < 0.001. Compared to the relationships reported in Fig 1 those calculated based on the average values of Ft and Fd have a slightly lower coefficient of determination and slope and a larger intercept. Thus, the choice to utilize the best values of Ft (largest) and Fd (smallest) indeed allows to refine our understanding of relationships between the power generated by thrust forces and the power needed to overcome drag force in sprint swimming (slopes closer to 1 and intercepts closer to 0, as it should be the case).

### General Discussion and Conclusions

The present study is the first to show that the swimmer’s thrust force is close to the force needed to overcome the swimmer’s drag in active conditions, as theoretically is the case. The relationship between the power generated by thrust forces and the power needed to overcome drag forces has indeed a large determination coefficient (R^2^ = 0.952), an intercept close to zero (1.09 W) and a slope close to the identity line (0.999); moreover, the difference between Pt and Pd_A_ is rather small (bias = -0.89 W), and the limits of agreement (-25.5 and 23.7 W) are about 50% of the SD of both Pd_A_ and Pt values (about 50 W).

Data were collected in three separate experiments, all referring to maximal sprint conditions: a maximal tethered test the duration of which (15 s) was as close as possible to that of the maximal swim test which, in turn, was covered at the same speed at which drag data were assessed/calculated.

The results of the present study should encourage coaches to consider the validity of the tethered test to evaluate the power balance of a swimmer. Indeed, when swimming at constant speed, propulsive forces should equal resistive forces and, therefore, to compute a swimmer’s power output either the power needed to overcome drag or the power generated by thrust forces could be measured. Whereas there is still a debate in the literature on how to measure power drag (and the method proposed in this study has its own weakness) our findings support the use of the tethered test as an accurate, reliable test, to determine the power balance in sprint swimming.

Indeed, since Fd·v = Ft·v, an increase in v without a proportional increase in Ft (in tests of similar duration and at maximal effort) would imply that the swimmer has reduced his/her hydrodynamic resistance (as could be the case when wearing a rubber body swimsuit, as an example) whereas an increase in v with a proportional increase in Ft would mean that Fd is unchanged, and so on. Thus the measure of the tethered force in relation to the swimmer’s maximal speed can help coaches monitoring the increase or decrease in the swimmer’s power output as well as to infer variations in his/her hydrodynamic resistance.
